# Like a Rolling Stone? A Review on Spontaneous Clearance of Hepatitis C Virus Infection

**DOI:** 10.3390/v16091386

**Published:** 2024-08-30

**Authors:** Piotr Rzymski, Michał Brzdęk, Krystyna Dobrowolska, Barbara Poniedziałek, Aleksandra Murawska-Ochab, Dorota Zarębska-Michaluk, Robert Flisiak

**Affiliations:** 1 Department of Environmental Medicine, Poznań University of Medical Sciences, 60-806 Poznań, Poland; bpon@ump.edu.pl; 2 Collegium Medicum, Jan Kochanowski University, 25-317 Kielce, Poland; michal.brzdek@gmail.com (M.B.); krystyna.dobrowolska98@gmail.com (K.D.); 3 Department of Infectious Diseases, Provincial Integrated Hospital in Kielce, 25-736 Kielce, Poland; almurawska@gmail.com; 4 Department of Infectious Diseases and Allergology, Jan Kochanowski University, 25-317 Kielce, Poland; dorota1010@tlen.pl; 5 Department of Infectious Diseases and Hepatology, Medical University of Białystok, 15-540 Białystok, Poland; robert.flisiak1@gmail.com

**Keywords:** viral hepatitis, viral clearance, viral genotypes, gene polymorphism, substance use, chronic infection

## Abstract

Elimination of hepatitis C virus (HCV) without the need for medical intervention, known as spontaneous clearance (SC), occurs at a significantly lower rate than in the case of hepatitis B virus infection and only in selected individuals, such as reportedly in Keith Richards, a guitarist of The Rolling Stones. The present paper provides an updated narrative review of the research devoted to the phenomenon in order to identify and discuss the demographic, lifestyle-related, clinical, viral genotype-related, and host genetic factors underpinning the SC occurrence. The body of evidence indicates that the likelihood of SC is decreased in older individuals, men, Black people, HIV-coinfected subjects, and intravenous drug and alcohol users. In turn, HBV coinfection and specific polymorphism of the genes encoding interferon lambda 3 (particularly at rs8099917) and interferon lambda 4 (particularly at rs12979860) and *HLA* genes increase the odds of SC. Numerous other host-specific genetic factors could be implicated in SC, but the evidence is limited only to certain ethnic groups and often does not account for confounding variables. SC of HCV infection is a complex process arising from a combination of various factors, though a genetic component may play a leading role in some cases. Understanding factors influencing the likelihood of this phenomenon justifies better surveillance of high-risk groups, decreasing health inequities in particular ethnic groups, and may guide the development of a prophylactic vaccine, which at present is not available, or novel therapeutic strategies. Further research is needed to elucidate the exact mechanisms underlying SC and to explore potential interventions that could enhance this natural antiviral response.

## 1. Introduction

Hepatitis C virus (HCV), a single-stranded RNA virus belonging to the Flaviviridae family discovered in 1989 [1,2], is primarily transmitted through blood-to-blood contact, such as sharing needles among people who inject drugs and through unsafe medical and cosmetic procedures. It can also be acquired sexually, especially among individuals with multiple sexual partners and men having sex with men, as well as vertically. Before the widespread screening, blood transfusion and receiving transplants also constituted a risk [3]. The virus has six major genotypes and numerous subtypes, contributing to its genetic diversity, geographical distribution, and variability in clinical presentation [4]. It primarily infects hepatocytes and leads to acute hepatitis, which is often asymptomatic or causes mild, nonspecific symptoms such as fatigue, fever, and abdominal discomfort, rarely jaundice, dark urine, and elevated liver enzymes. If progressing to chronic hepatitis, it frequently remains asymptomatic for years or even decades, causing delayed diagnosis and treatment initiation, eventually leading to severe liver complications such as fibrosis, cirrhosis, hepatocellular carcinoma, and end-stage liver disease. The World Health Organization estimated that approximately 58 million people worldwide were living with chronic HCV infection in 2022 [5]. Advances in diagnostics and treatment, especially the introduction of direct-acting antivirals, have transformed the management of hepatitis C, leading to improved outcomes and the potential to significantly reduce liver-related mortality [6,7,8].

Some individuals, however, experience spontaneous clearance (SC) of HCV infection during the acute phase without a need for antiviral treatment. As Keith Richards, a guitarist of The Rolling Stones, who reportedly experienced SC, put it in *Life*, his memoir: “*I cured myself of hepatitis C without even bothering to do anything about it*”. Such a phenomenon of viral elimination can occur for at least 12 months following the infection [9] (with some studies reporting extended periods [10]) and is associated with a favorable prognosis, reduced risk of liver fibrosis progression, and improved long-term outcomes compared with chronic infection. How often it occurs is a subject of ongoing discussion and research, with some studies suggesting that it might be significantly underestimated due to challenges stemming from difficulties in identifying and tracking acute infections, the heterogeneity of host and viral factors, reinfection risks, and the complexities involved in longitudinal follow-up of high-risk populations [11].

Understanding the exact factors contributing to this phenomenon is also an active field of research for several reasons. It may inform the development of novel therapeutic strategies, especially for those who still experience failure with conventional antiviral treatment regimes [12,13]. It can also support the progress of works on the prophylactic vaccine, the development of which remains challenging but the availability of which is likely essential to eventually put HCV under improved control, especially in high-risk groups [14,15]. Understanding SC rates and factors influencing it can also inform the natural history of the disease, aiding in epidemiological models that help shape public health policies for screening, treatment, and prevention. Last but not least, it can expand knowledge of host–virus interactions, some of which are still poorly elucidated [16]. This information could help strategize individuals who are more likely to progress to chronic infection, thus enabling targeted interventions for those at higher risk.

Therefore, in the present paper, we provide an updated narrative review of the studies on the incidence of HCV SC in different world regions and discuss the factors that favor and do not favor its occurrence, including demographic, lifestyle-associated, host genetic, immunological, clinical, and viral genotype-related. To this end, a semi-quantitative approach based on a Boolean search of the PubMed and Scopus databases was conducted using a search string with terms accounting for HCV and spontaneous clearance to identify English language publications published in peer-reviewed journals between 2000 and April 2024. The retrieved records were pre-screened by title and abstract, and only original research articles were included. In total, 55 articles reporting on studies on factors influencing SC of HCV were identified and used in this review paper.

## 2. Prevalence of Spontaneous HCV Clearance

SC of HCV is reported with a frequency ranging from a few percent to over 70%. This large discrepancy in the incidence of SC is determined by the heterogeneity of the analyzed populations in terms of size, the distribution of positive and negative predictors of this phenomenon, length of the observation period, and methodology. It is also important to determine whether SC rates were assessed in patients after acute HCV infection or in patients with established chronic infection. According to data available in the literature, in the case of newly acquired HCV infection, the chances of SC of the virus are much higher than in the case of chronic infection [17]. A meta-analysis of 31 prospective longitudinal studies involving 675 patients carried out by Micaleff et al. estimated the average chance of HCV clearance following acute infection at about 25% [18]. In general, prospective studies consistently report higher clearance than retrospective population-based studies (which usually rely on anti-HCV antibody positivity and HCV RNA negativity among people who do not have a recorded history of treatment). Additionally, testing frequency is important; among those with ongoing risk factors, such as people who inject drugs, some episodes of SC may be missed if reinfection occurs between testing intervals.

In a prospective study involving a population of young intravenous drug users who were not infected with HCV conducted in California between 2000 and 2007, 135 newly acquired HCV infections were identified; among 95 patients with available follow-up data, 21% achieved spontaneous viral clearance [19]. A comparable rate of SC (21.6%) was documented in a pooled analysis of observational studies of intravenous drug users from the US, Canada, Australia, and the Netherlands, including a total of 411 patients with acute HCV infection [20].

A Danish cohort of people living with HIV showed a 23% prevalence of SC after acute HCV infection, and importantly, among HBV-coinfected individuals, the rate reached as high as 65% [21]. In a population of 214 Italian patients infected with HCV mainly through the use of intravenous drugs and medical procedures between 1999 and 2004, of whom 68% had symptomatic acute hepatitis, the percentage of those with SC was 36% [22]. An Irish retrospective study assessing the clinical consequences of HCV infection through administering an infected anti-D immunoglobulin documented a 45% clearance rate in a population of 704 women; the female gender may provide a reasonable explanation for this high percentage of SC [23]. An even higher rate of spontaneous HCV clearance of up to 63% was described in Egyptian patients with iatrogenic genotype 4 infection; of the 19 patients who experienced SC, 15 were women [24].

An interesting finding regarding the doubling of SC prevalence from 32.6% to 68.7% over the years comes from an observational study comparing annual clearance rates in the period 1998–2017 among patients infected with HCV in California [25]. The authors suggest that earlier patients were more often screened for diagnostic purposes or for known risk factors rather than as true screening as in later years. The strength of this analysis is the considerable population size of more than 25,000 participants.

Although HCV SC is more common following acute HCV infection, it is also possible once chronic infection is established. Many reports of this phenomenon are based on case reports or case series describing this situation in patients under exceptional circumstances, such as withdrawal of immunosuppressive treatment during pregnancy or after childbirth [26,27]. Cases of SC are described even in advanced stages of liver disease in cirrhotic patients after splenectomy or liver transplantation [28,29]. There are also reports of spontaneous HCV clearance in patients with chronic hepatitis C after acute HBV or HBV/HDV superinfection, probably as a consequence of the robust and multispecific CD4^+^ and CD8^+^ T-cell responses documented in these cases [30,31,32]. This suggests that HCV can be eliminated through bystander immunological processes triggered by acute infections with other viruses.

In addition to case reports, there is evidence of SC in chronic HCV infection from population-based studies reporting it at 0.3–0.7% per person-year. A long-term cohort study conducted in Japan involving 435 chronically HCV-infected patients followed for an average of seven years documented spontaneous viral clearance in 16 patients (3.9%) with a rate of 0.5/person-year [33]. A nearly identical result of 3.6% during the same 7-year follow-up period was documented in a cohort of 139 Alaska Natives, in which 5 experienced sustained HCV clearance; it is noteworthy that all of them were women [34].

Quite a few reports of well-documented spontaneous HCV clearance in chronic infection come from observations of HIV-infected populations, likely due to the detailed surveillance of these patients during antiretroviral therapy. In addition to case reports, analyses from large real-world experience study cohorts of patients with HIV/HCV coinfection are available [35,36,37]. A retrospective analysis of records from 1994 to 2013 of 10,318 HIV/HCV coinfected patients from the West of Scotland identified 50 individuals who had spontaneous HCV clearance corresponding to 0.36/person-year [38]. The phenomenon was more frequent in women, those who acquired the infection at a younger age, patients with lower HCV viral loads, and HBV-coinfected individuals. The possibility of spontaneous HCV clearance in chronic infection in people living with HIV was also demonstrated in the ESPRIT phase III clinical trial evaluating the effect of adding interleukin-2 to antiretroviral therapy, in which SD assessment was a secondary endpoint of analysis. Among HIV-infected participants, 312 patients were chronically infected with HCV, and nine of them (2.9%) experienced SC during the study and the 5-year follow-up period [39]. In turn, the European cohort studies of recently acquired HCV infections in HIV-positive men who have sex with men found a 12–14% rate of SC [40,41]. Considering that antiretroviral drugs do not have direct activity against HCV, the reconstitution of the immune response may be responsible for the SC of the virus in the phase of chronic infection.

## 3. Factors Influencing the Occurrence of HCV Spontaneous Clearance

### 3.1. Demographic Factors

There are several demographic parameters influencing the odds of SC of HCV infection. Firstly, studies have shown that this phenomenon is more likely to occur at a younger age (Table 1). Three main factors may contribute to this age-related difference: (i) immunosenescence in older individuals translating in diminished immune surveillance and response, (ii) co-morbidities, which are more likely to be present in older individuals and may further complicate the immune response and affect the liver function, and (iii) changes in hepatic function (e.g., increased liver fibrosis) accumulated with age and affecting the response to HCV infection.

The female sex has also been consistently associated with a higher rate of SC of HCV infection compared with males (Table 1). Several factors, including hormonal influences, immune response differences, and behavioral and environmental characteristics, contribute to this observed disparity. Firstly, estrogen is known to reveal anti-inflammatory effects and modulate immune activity, supporting viral clearance. Notably, 17β-estradiol induces an interferon-mediated antiviral state in hepatocytes, perturbing HCV’s assembly and release, modulating viral entry, and ultimately, suppressing an infection [42,43,44,45]. In addition, it has also been shown to have protective effects on the liver, including reducing fibrosis and promoting hepatic regeneration, potentially increasing the likelihood of SC [46,47]. Secondly, women generally exhibit a more robust immune response than males, i.e., a higher number of CD^4+^ T cells [48], which play a leading role in controlling HCV infection. Their failure is a hallmark that increases the likelihood of progression to chronic hepatitis C [49]. Conversely, increased CD4+ T cells may translate into higher odds of SC of HCV infection in women. Thirdly, females may have different risk profiles or exposure to co-factors that affect the likelihood of SC, such as intravenous substance use [50] and alcohol consumption [51]. However, one should note that this predominantly applies to premenopausal women as the frequency of HCV infections and progression to unfavorable clinical consequences in the postmenopausal period is significantly increased [52].

**Table 1:** The summary of analyses evidencing significant associations between demographic characteristics and the likelihood of spontaneous clearance of HCV infection.

Region	Participants	OR of Spontaneous Clearance (95% CI)	Reference
**Age**			
**USA**	*n* = 101 aged ≥ 18	Age < 30 *vs*. ≥30 2.97 (1.98–4.46) MA	[53]
**China**	*n* = 347 aged ≥ 19	Age < 25 *vs*. ≥25 2.50 (1.10–5.65) MA	[54]
**USA**	*n* = 712 aged ≥ 13	Age 16–60 *vs*. <2 0.34 (0.18–0.65) MA	[55]
**USA**	*n* = 420 aged ≥ 18	<50 *vs*. ≥50 5.0 (1.1–10.0) UA	[56]
**Latvia**	*n* = 61 (children and adults)	Children *vs*. adults 3.50 UA	[57]
**Slavic population**	*n* = 367 aged ≥18	18–35: 2.30 (1.24–4.29) UA 36–45: 0.43 (0.20–0.92) UA	[58]
**China**	*n* = 498 median age 34, PWIDs	Age < 40 *vs*. ≥40 2.8 (1.0–7.7) UA	[59]
**Female *vs.* male sex**			
**USA**	*n* = 919 aged ≥ 17	1.58 (0.98–2.54) UA	[17]
**Denmark**	*n* = 327 median age (IQR) 36 (30–41)	1.8 (1–3.2) MA	[21]
**Netherlands**	*n* = 106	6.62 (2.69–26.13) MA	[60]
**Egypt**	n = 4720 aged ≥ 18	1.59 (1.21–2.08) MA	[61]
**China**	*n* = 410 mean age (SD) 50.6 (9.1)	2.18 (1.13–4.21) MA	[62]
**China**	*n* = 402 mean age (SD) 53.7 (7.4)	1.98 (1.12–3.34) MA	[63]
**Europe, Israel, Argentina**	*n* = 1940 median years of age 37.2	1.39 (1.06–1.81) MA	[64]
**Japan**	*n* = 993 aged ≥ 30	2.27 (1.16–4.45) MA	[65]
**Slavic population**	*n* = 367 aged ≥ 18	2.723 (1.460–5.079) UA	[58]
**Canada**	*n* = 762	1.6 (1.1–2.4) MA	[66]
**Black *vs.* non-Black**			
**USA**	*n* = 919 aged ≥ 17	0.19 (0.01–0.38) MA	[17]
**USA**	*n* = 712 aged ≥ 13	0.46 (0.23–0.91) MA	[55]
**USA**	*n* = 320 aged ≥ 18	0.43 (0.21–0.92) MA	[67]
**USA**	*n* = 695 aged <20–>60	0.37 (0.20–0.70) MA	[68]
**USA**	*n* = 897 aged ≥ 18	HIV-positive 0.57 (0.36–0.93) UA	[69]
		HIV-negative 0.26 (0.09–0.79) MA	
**USA**	*n* = 302 aged ≥ 21	0.11 (0.01–0.87) MA	[70]
**USA**	*n* = 420 (aged ≥ 18)	*vs*. Caucasian 0.32 (0.15–0.67) MA *vs*. Hispanic/Other 0.29 (0.12–0.77) MA	[56]

IQR—interquartile range; MA—multivariate analysis; OR—odds ratio; PWIDs—people who inject drugs; SD—standard deviation; UA—univariate analysis.

Moreover, research has suggested that ethnicity may influence the likelihood of HCV SC, with black individuals generally exhibiting lower rates compared with individuals of other ethnic backgrounds (Table 1). This discrepancy can be attributed to a combination of genetic and environmental factors. A specific favorable *IFNL4* polymorphism, i.e., rs12979860, which has been found to correlate with a higher rate of SC, is less common in individuals of African descent compared with those of European descent, contributing to lower clearance rates among Black people [71,72]. Moreover, disparities in healthcare access and quality, resulting in higher rates of certain conditions, such as obesity, diabetes, or hypertension [73], can affect the liver and immune system, ultimately reducing the likelihood of SC of HCV infection in Black individuals.

### 3.2. Use of Psychoactive Substances

Studies show that substance use can affect the rate of SC. The majority of them indicate that intravenous drug use (IDU) can affect these rates negatively when controlling for confounding variables (Table 2). However, there are challenges to accurately measuring SC in people who use intravenous drugs due to the loss in follow-up [74,75,76]. Nevertheless, it is known that opioids, which are predominant among drugs administrated intravenously, have immunomodulatory effects and can blunt certain immune responses, including altering the cytotoxic activity of natural killer cells [77], lymphocyte activity [78] and cytokine production [79], all of which can contribute to progression to chronic HCV infection [80,81]. This clearly indicates that people with a history of IDU should be a target of HCV diagnostics as they may require treatment more frequently than other groups exposed to this virus. The evidence that non-IDU can impact HCV SC has been explored much less frequently. In one study, illicit drug use (both among IDU and non-IDU) was associated with lower odds of SC, but no specific association with non-IDU was found [66].

In addition, several studies suggest that abstaining from alcohol may increase the likelihood of HCV SC and that alcohol use is generally associated with reduced clearance when controlling for various confounding variables (Table 2). There are several pathways behind this phenomenon, including alcohol-induced diminishing of immune responses by altering dendritic cell function against particular viral proteins (i.e., NS5) by reducing expression of CD40 and CD86, impairing allostimulatory activity and altering cytokine expressions, ultimately resulting in decreased CD8^+^ T-cell activity necessary for viral clearance. [82] Acetaldehyde, a metabolite of ethanol, was also shown to suppress interferon-alpha signaling in hepatic cells by enhancing HCV-induced suppression of STAT-1 methylation [83]. Moreover, ethanol exposure can increase cellular oxidative stress, the risk of hepatic steatosis, and the rate of hepatocyte apoptosis, all likely contributing to higher odds of failure of HCV SC [84]. Interestingly, the negative effect on viral clearance may be more profound in women compared with men [20], which is an important observation given that the former are generally more likely to experience SC (Table 1), while the latter are consuming more alcohol on average, though there is a recent increase in this regard in women [51,85]. Of note, there is one case report suggesting that increased alcohol consumption may have played a role in the SC of HCV genotype 3a in men with chronic HCV infection [86]. As speculated, such effect may occur through alcohol-induced enhanced stimulation of TRL2 and TLR4 receptors, activation of the NF-kB pathway, and dysregulation of cytokine profile, though an exact mechanism remains not well understood, and such a phenomenon shall be treated, at best, as rare. Therefore, it is reasonable to conclude that abstaining from alcohol and managing alcohol use disorder may be beneficial for viral clearance, be it spontaneous or guided by DAA treatment [87].

**Table 2:** The summary of analyses evidencing significant associations between substance use and the likelihood of spontaneous clearance of HCV infection.

Region	Participants	OR of Spontaneous Clearance (95% CI)	Reference
**Intravenous drug use**			
**USA**	*n* = 6890 aged ≥ 17	0.40 (0.28–0.58) MA	[88]
**Denmark**	*n* = 327 median age (IQR) 36 (30–41)	5.2 (1.2–23.5) MA	[21]
**Europe, Israel, Argentina**	*n* = 1940 median years of age 37.2	0.36 (0.24–0.53) MA	[64]
**Canada**	*n* = 762	0.54 (0.29–1.00) MA	[66]
**Iran**	*n* = 50,045 aged ≥ 40	3.27 (1.78–6.00) MA	[89]
**Switzerland, Italy**	*n* = 1450 median age (IQR) spontaneous clearance 8 (19) median age (IQR) chronic infection 20 (9)	vs. invasive procedure, needle stick 0.62 (0.38–1.02) MA vs. other/missing 0.28 (0.17–0.46) MA	[90]
**Non-intravenous drug use**			
**Iran**	*n* = 50,045 aged ≥ 40	1.90 (1.07–3.39) MA	[89]
**Alcohol use**			
**USA**	*n* = 496 aged <50–>50	0.49 (0.30–0.81) MA	[91]
**USA**	*n* = 302 aged ≥21	>0–<2 drinks per week 2.29 (1.05–5.00) MA	[70]
		2–<7 drinks per week 2.62 (1.22–5.62) MA	[70]
		>7 drinks per week 2.39 (1.13–5.03) MA	[70]
**USA**	*n* = 101 aged ≥ 18	≥14 drinks per week 0.51 (0.27–0.98) UA	[53]

IQR—interquartile range; MA—multivariate analysis; OR—odds ratio; UA—univariate analysis.

### 3.3. Genetic Factors

There is substantial evidence that host polymorphisms play a significant role in determining the outcome of the HCV infection, including the likelihood of SC [92]. The summary of research revealing the odds of SC in relation to various genetic predispositions is given in Table 3. Key polymorphisms shown to affect the natural course of HCV infection and associated with SC of HCV include the following:

- Alleles of human leukocyte antigen (*HLA*) genes, including HLA-C*01 and HLA class II alleles, such as certain alleles of HLA-DQB1*01, HLA-DQB1*02, HLA-DQB1*04, HLA-DQB1*11, HLA-DQB1*12, and HLA-DQB1*14, have been associated with enhanced HCV clearance, possibly due to their ability to present viral antigens effectively to cytotoxic T cells. In turn, HLA-C*05, HLA-DQB1*02, and HLA-DQB1*07 decreased the odds of SC (Table 3). One should note that the association between certain alleles has been tested only in particular ethnic groups, e.g., HLA-DQB1*16:01 and HLA-DQB4*01:01 were shown to increase and decrease the odds of SC, respectively, only in the Caucasian population. Some HLA alleles likely play a role in SC only in particular populations, e.g., HLA-DRB1*07, for which the positive association was only evidenced in Caucasians [92]. Importantly, the majority of conducted studies employed univariate analyses without controlling for confounding variables, including demographic, substance use, and clinical parameters (Table 4)
- Polymorphisms in the genes encoding interferons-lambda, IFN-λ3 (*IFNL3*), and IFN-λ4 (*IFNL4*), belonging to the type III interferon group, have been strongly linked to the SC of HCV infection (Table 4 and Table 5). These molecules are known to reveal antiviral properties mediated through the stimulation of the Janus kinase signal transducer and activator of transcription (protein, which controls interferon (interferon-stimulated genes) [93]. Particularly, the C/CC genotypes at rs12979860 of *IFNL4* have been consistently associated with increased odds of SC in various populations (Table 5). There is also substantial evidence that G/GG genotypes at rs4803217 of *IFNL3* increase rate can independently increase the likelihood of HCV SC (Table 4). Several other polymorphisms of the *IFNL3* gene were also found to be potentially associated with SC, e.g., genotype AA at rs11881222, T genotype at rs8103142, C/CC genotype at rs4803222, T genotype at rs4803219, G genotype at rs28416813, TT genotype at rs8105790, CC genotype at rs10853728, and AA genotype at rs35790907, but the evidence is limited only to certain populations, the analyzed groups were relatively small or the analysis did not account for confounding variables (Table 4). Similarly, there are various other polymorphisms of the *IFNL4* gene, possibly increasing the likelihood of SC, but further confirmation in multivariate analysis encompassing different ethnic groups would be necessary (Table 5).
- Polymorphism of other genes, including gene encoding paraoxonase 1 (*PON1*), Signal transducer CD24 (*CD24*), receptor retinoic acid-related orphan receptor C (*RORC*), and MX dynamin-like GTPase (*MX1*) (Table 6). *CD24* encodes for glycosylphosphatidylinositol-anchored cell-surface glycoprotein that is expressed by various immune cells and plays an important role in inflammation and regulates innate and adaptive immune responses [94,95,96]. Paraoxonase-1 is a high-density lipoprotein-associated esterase with the capability to prevent lipid oxidation, with certain *PON1* variants related to anti-inflammatory and anti-thrombosis processes [97,98,99]
- In turn, *RORC* is a transcription factor regulating Th17 differentiation, a subset of pro-inflammatory T cells that play an antiviral role through the secretion of different cytokines (IL-17, IL-21, and IL-22) and responses of which are thought to be associated with SC [100,101,102]. *MX1* encodes a guanosine triphosphate (GTP), a metabolizing protein induced by type I and type II interferons, and antagonizes viral replication [103]. Therefore, all of these genes may be engaged, to some extent, in responses against HCV infection, with their polymorphism potentially affecting these capabilities. One should note that the existing evidence behind it is currently scarce, limited to certain ethnical groups, encompassing relatively small groups, and sometimes without control over various confounding variables. Therefore, it is encouraged to pursue further studies in this regard that would improve the understanding of whether polymorphism of these genes is an independent predictor of SC.

**Table 3:** The summary of analyses evidencing significant associations between *HLA* gene polymorphism and the likelihood of spontaneous clearance of HCV infection.

Gene Polymorphism	Region	Participants	OR (95% CI) of Spontaneous Clearance	Reference
HLA-C*01:02	Global	1433 patients, diverse population	2.35 (1.11–4.93) UA	[92]
HLA-C*05:00		784 Caucasians	0.67 (0.46–0.98) UA	
HLA-DQB1*02:00		694 Caucasians	0.35 (0.22–0.57) UA	
		1623 patients, diverse population	0.49 (0.30–0.82) UA	
HLA-DQB1*02:01		927 Caucasians	0.61 (0.45–0.83) UA	
		100 Asians	0.13 (0.03–0.58) UA	
HLA-DQB1*03:00		722 Caucasians	2.94 (1.42–6.11) UA	
		1053, diverse population	2.55 (1.71–3.80) UA	
HLA-DQB1*03:01		1764 Caucasians	2.04 (1.23–3.38) UA	
		100 Asians	2.74; (1.20–6.22) UA	
		3206, diverse population	1.93 (1.34–2.77) UA	
	China	143 Chinese aged 60 to 92	3.90 (1.27–11.95) MA	[104]
HLA-DQB1*01:01	Global	2403, diverse population	1.66 (1.02–2.70) UA	[92]
HLA-DQB1*04:00		893 Caucasians	1.71 (1.10–2.64) UA	
		1683 diverse population	1.66 (1.22–2.26) UA	
HLA-DQB1*07:00		512 Caucasians	0.51 (0.30–0.87) UA	
HLA-DQB1*07:01		602 Caucasians	0.47 (0.31–0.72) UA	
HLA-DQB1*11:00		763 Caucasians	2.12 (1.26–3.57) UA	
		1094 diverse population	1.88 (1.32–2.66) UA	
HLA-DQB1*11:01		1023 Caucasians	2.66 (1.90–3.70) UA	
		2615 diverse population	1.87 (1.21–2.90) UA	
	China	143 Chinese aged 60 to 92	3.38 (1.02–11.27) UA	[104]
HLA-DQB1*12:01	Global	160 Hispanic	7.15 (1.70–30.1) UA	[92]
		1482 diverse population	2.47 (1.09–5.56) UA	
HLA-DQB1*13:03		591 Caucasians	3.68 (1.15–11.80) UA	
HLA-DQB1*14:00		1084 diverse population	2.24 (1.05–4.75) UA	
HLA-DQB1*16:01		464 Caucasians	2.71 (1.13–6.52) UA	
HLA-DQB4*01:01		464 Caucasians	0.47 (0.31–0.72) UA	
**HLA-DQB3** **rs4273729** **CC genotype**	China	1118 Chinese (Han population) ≥18 years old	CC *vs*. GG 0.37 (0.23–0.58) MA	[105]

MA—multivariate analysis; OR—odds ratio; UA—univariate analysis.

**Table 4:** The summary of analyses evidencing significant associations between polymorphism of the *IFNL3* gene and the likelihood of spontaneous clearance of HCV infection.

Gene Polymorphism	Region	Participants	OR (95% CI) of Spontaneous Clearance	Reference
**rs4803217** **G allele**	Egypt	261 Egyptians aged 2–88	2.68 (1.62–4.41) UA	[106]
	Switzerland	389 HIV/HCV-coinfected individuals aged ≥ 18	3.0 (2.0–4.6) MA	[107]
	Ireland	71 women aged 34–60	4.1 (1.4–11.8) MA	
**rs4803217** **GG genotype**	Poland	161 Polish hemodialyzed patients aged 9–80	GG *vs*. GT + TT 2.52 (1.32–4.84) UA	[108]
	USA	1475 African Americans aged ≥18	GG *vs*. TT 3.22 (2.20–4.72) UA	[109]
	USA	611 European Americans aged ≥18	GG *vs*. TT 5.02 (2.08–12.10) UA	
	Taiwan	889 patients aged 30–65	GG *vs*. GT + TT 2.55 (1.42–4.6) MA	[110]
**rs4803217** **TT genotype**	Italy	167 children aged > 30 months	TT *vs*. GT 0.12 (0.02–0.6) * UA	[111]
**rs11881222** **AA genotype**	Taiwan	889 patients aged 30–65	AA *vs*. AG + GG 2.54 (1.41–4.58) MA	[110]
**rs12980275** **A allele**	Egypt	261 Egyptians aged 2–88	2.87 (1.88–4.40) UA	[106]
**rs12980275** **AA genotype**	Poland	161 Polish hemodialyzed patients aged 9–80,	AA *vs*. AG + GG 2.8 (1.45–5.43) UA	[108]
	China	376 Chinese, mean age (SD): 53.2 (8) years	AA *vs*. AG 7.92 (1.88–33.32) UA	[112]
	Taiwan	889 patients aged 30–65	AA *vs*. AG + GG 2.23 (1.27–3.91) MA	[110]
**rs8103142** **T allele**	Egypt	261 Egyptians aged 2–88 years	2.76 (1.84–4.13) UA	[106]
	Switzerland	389 HIV/HCV-coinfected individuals aged ≥18	3.0 (2.0–4.6) MA	[107]
	Ireland	71 women aged 34–60	4.1 (1.4–11.8) MA	
**rs4803222** **C allele**	Egypt	261 Egyptians aged 2–88	2.61 (1.67–4.06) UA	[106]
**rs4803222** **CC genotype**	Taiwan	889 patients aged 30–65	CC *vs*. CG + GG 2.54 (1.41–4.58) MA	[110]
**rs4803219 T allele**	Switzerland	389 HIV/HCV-coinfected individuals aged ≥18	2.6 (1.7–3.8) MA	[107]
	Ireland	71 women aged 34–60	4.6 (1.6–13.6) MA	
**rs28416813 G allele**	Switzerland	389 HIV/HCV-coinfected individuals aged ≥18	3.0 (1.9–4.5) MA	
	Ireland	71 women aged 34–60	4.1 (1.4–11.8) MA	
**rs8105790 TT genotype**	China	376 Chinese, mean age (SD): 53.2 (8)	TT *vs*. CT 14.88 (2.02–109.72) UA	[112]
**rs10853728** **CC genotype**	China	376 Chinese, mean age (SD): 53.2 (8)	CC *vs*. CG + GG 2.32 (1.22–4.42) UA	
**rs35790907** **AA genotype**	Taiwan	889 patients aged 30–65	AA *vs*. AT + TT 2.35 (1.37–4.04) MA	[110]

* 90% confidence interval, range; MA—multivariate analysis; OR—odds ratio; SD—standard deviation; UA—univariate analysis.

**Table 5:** The summary of analyses evidencing significant associations between polymorphism of the *IFNL4* gene and the likelihood of spontaneous clearance of HCV infection.

Gene Polymorphism	Region	Participants	OR (95% CI) of Spontaneous Clearance	Reference
**rs12979860** **C allele**	Italy	167 children aged > 30 months	2.3 (1.4–3.9) * UA	[111]
	Italy	177 children aged > 30 months	2.5 (1.4–4.6) * UA	[113]
	Ireland	543 Women, Irish Caucasians, aged 16–44	4.20 (3.05–5.79 UA	[114]
	Egypt	162 North Africans with GT4	2.30 (1.40–3.80) UA	[115]
	Egypt	261 Egyptians aged 2–88	2.84 (1.87–4.30) UA	[106]
	Switzerland	389 HIV/HCV-coinfected individuals aged ≥ 18	3.0 (1.9–4.5) MA	[107]
	Ireland	71 women aged 34–60	4.1 (1.4–11.8) MA	
**rs12979860** **CC genotype**	Ireland	543 Women, Irish Caucasians, aged 16–44	CC *vs*. CT + TT 7.38 (4.93–11.07) UA	[114]
	Ireland	543 Women, Irish Caucasians, aged 16–44	CC *vs*. TT 8.76 (3.26–24.82) UA	
	Egypt	162 North Africans with GT4	CC *vs*. TT 3.8 (1.3–11.5 UA	[115]
	UK	323, >90% Caucasians	2.97 (1.76–5.00) UA	[116]
	Italy	177 children aged >30 months	CC *vs*. CT + TT: 2.7 (1.3–5.8) * UA	[113]
	Egypt	162 North Africans with GT4	CC *vs*. TT 3.3 (1.7–6.6) UA	[115]
	Italy	177 children aged >30 months	CC *vs*. TT 10.3 (1.3–217.8) * UA	[113]
	Egypt	162 North Africans with GT4	CC *vs*. CT + TT 3.4 (1.8–6.5) UA	[115]
	Italy	167 children aged > 30 months	CC *vs*. CT + TT 2.3 (1.2–4.5) * UA	[111]
	Italy	167 children aged > 30 months	CC *vs*. TT 10.6 (1.8–60.8) * UA	
	Poland	161 Polish hemodialyzed patients aged 9–80	CC *vs*. CT + TT: 2.44 (1.27–4.69) UA	[108]
	Various	7363 patients, diverse population	CC *vs*. CT + TT 2.75 (2.23–3.38) UA	[117]
	USA	555 African American, median age (IQR) 40.7 (37.2–44.3)	CC *vs*. TT 2.75 (1.49–5.08) UA	[118]
	USA	459 African Americans, aged ≥18, IDUs	CC *vs*. TT 2.77 (1.48–5.17) UA	
	USA		CC *vs*. TT 4.76 (2.40–9.43) UA	
	USA	245 thalassemia major patients	CC *vs*. TT 2.42 (1.44–4.09) UA	[119]
	USA, UK	642 patients of European ancestry	CC *vs*. TT 2 (1.02–4.0) MA	[72]
	USA, UK	290 patients of African ancestry	CC *vs*. TT 4.76 (2.27–10) MA	
	Germany	190 women, mean age (SD) 24.6 (4) years	CC *vs*. TT 27.9 (6.1–126.3) UA	[120]
	Germany	396 Caucasian women, mean age (SD) 24.7 (4) years, GT1	CC *vs*. CT 3.7 (2.3–6.0) UA	[121]
	USA, UK	Subjects of European ancestry	CC *vs*. CT 2.78 (1.92- 4.17) MA	[72]
	USA, UK	Subjects of African ancestry	CC *vs*. CT 2.5 (1.33–4.76) MA	
	Germany	190 women, mean age (SD) 24.6 (4)	CC *vs*. CT 5.54 (2.77–11.07) UA	[120]
	India	557 patients with thalassemia aged 1.9–37	CC *vs*. CT + TT 3.87 (2.57–5.83) UA	[122]
	Italy	28 caucasian children	CC *vs*. CT + TT 15 (1.2–376) UA	[123]
	Spain	353 Spanish population	CC *vs*. CT + TT 3.13 (1.72–5.56) UA	[112]
	Brazil	138 Brazilian patients with HIV-1, mean age (SD): 41.7 (9.5)	CC *vs*. CT + TT 2.78 (1.16–6.64) UA	[124]
	Italy	149 Caucasian with thalassemia	CC *vs*. CT + TT 4.1 (1.7–9.2) MA	[125]
	Germany	396 Caucasian women, mean age (SD) 24.7 (4) years, GT1	CC *vs*. CT + TT 4.2 (2.7–6.7) UA	[121]
	China	725 Chinese (Han population)	CC *vs*. CT + TT 2.12 (1.01–4.42) MA	[126]
	USA, UK	Subjects of European ancestry	CC *vs*. CT + TT 2.63 (1.85–3.85) MA	[72]
	USA, UK	Subjects of African ancestry	CC *vs*. CT + TT 3.12 (1.75–5.88) MA	
	Germany	190 women, mean age (SD) 24.6 (4)	CC *vs*. CT + TT 7.39 (3.78–14.43) UA	[120]
	Taiwan	889 patients aged 30–65	CC *vs*. CT + TT 2.65 (1.47–4.76) MA	[110]
	USA	664 African Americans aged ≥18 IDUs	CT *vs*. TT 2.05 (1.09–3.82) UA	[118]
	Germany	190 women, mean age (SD) 24.6 (4)	CT *vs*. TT 5.01 (1.11–22.67) UA	[120]
	UK	323, >90% Caucasians	0.35 (0.20–0.60) UA	[116]
**rs8109889** **CC genotype**	Taiwan	889 patients aged 30–65	CC *vs*. CT + TT 2.53 (1.38–4.63) MA	[110]
**rs8113007** **TT genotype**	Taiwan		TT *vs*. TA + AA 2.36 (1.33–4.21) MA	
**rs73050457** **CC genotype**	Taiwan		CC *vs*. CT + TT 3.12 (1.41–6.87) MA	
**rs368234815** **TT genotype**	Italy	167 children aged > 30 months	2.5 (1.5–4.3) * UA	[111]
**rs368234815** **TT/TT genotype**	Italy		TT/TT *vs*. ΔG/TT + ΔG/ΔG 2.8 (1.4–5.5) * UA	
	Poland	187 hemodialyzed patients	TT/TT *vs*. ΔG/TT + ΔG/ΔG 2.6 (1.3- 5.2) UA	[97]
	Italy	167 children aged >30 months	TT/TT *vs*. ΔG/ΔG 12.6 (2.2–72.3) *, UA	[111]
	USA	1475 African American, ≥18	TT/TT *vs*. ΔG/ΔG 4.14 (2.81–6.11) UA	[109]
	Poland	161 Polish hemodialyzed patients aged 9–80	TT/TT *vs*. ∆G/TT + ∆G/∆G 2.63 (1.38–5.04) UA	[108]
	Taiwan	889 patients aged 30–65	TT/TT *vs*. ∆G/TT + ∆G/∆G 2.85 (1.48–5.46) UA	[110]
**rs368234815** **ΔG/TT genotype**	USA	611 European American, ≥18	ΔG/TT *vs*. ΔG/ΔG 5.58 (2.32–13.41) UA	[109]
**rs8099917** **T allele**	Egypt	261 Egyptians aged 2–88	2.61 (1.51–4.49) UA	[106]
	Switzerland	389 HIV/HCV-coinfected individuals aged ≥18	2.5 (1.6–3.9) UA	[107]
	Ireland	71 women aged 34–60	3.3 (1.0–11.2) MA	
**rs8099917** **TT genotype**	China	143 Chinese aged 60 to 92	TT *vs*. TG): 10.58 (1.25, 89.34)	[104]
	China	376 Chinese, aged mean (SD): 53.2 (8)	TT *vs*. GT 15.27 (2.07–112.50) UA	[112]
	Poland	161 Polish hemodialyzed patients aged 9–80,	TT *vs*. GT + GG 2.75 (1.39–5.45) UA	[108]
	India	557 patients with thalassemia aged 1.9–37	TT *vs*. GT + GG 1.66 (1.09–2.53) UA	[122]
	USA	245 thalassemia major patients	TT *vs*. GT + GG 2.13 (1.22–3.73) UA	[119]
	Switzerland, Germany	1362 white	TT *vs*. GT + GG 4.79 (1.68–13.67) MA	[127]
	Taiwan	889 patients aged 30–65 years	TT *vs*. GT+ GG 2.42 (1.29–4.53) MA	[110]
**ss469415590** **TT/TT genotype**	USA	555 women, African American, median age (IQR) 40.7 (37.2–44.3)	TT/TT *vs*. ΔG/ΔG 3.59 (1.96–6.56) UA	[118]
	USA	185 women, Hispanic, median age (IQR) 36.8 (32.7–41.0)	TT/TT *vs*. ΔG/ΔG 6.52 (2.04–20.88) UA	
	USA	150 women, White, median age (IQR) 36.9 (32.9–42.0)	TT/TT *vs*. ΔG/ΔG 12.06 (1.90–∞) UA	
	USA	459 African Americans, aged ≥18, PWIDs	TT/TT *vs*. ΔG/ΔG 3.51 (1.88–6.56) UA	
	USA	664 aged ≥ 18, African American, PWIDs	TT/TT *vs*. ΔG/ΔG 4.68 (2.40–9.10) UA	
	USA	664 aged ≥ 18 African American, PWIDs	TT/ΔG *vs*. ΔG/ΔG 1.82 (1.02–3.26) UA	

* 90% confidence interval, GT—genotype; IQR—interquartile range; MA—multivariate analysis; OR—odds ratio; PWIDs—people who inject drugs; SD—standard deviation; UA—univariate analysis.

**Table 6:** The summary of analyses evidencing significant associations between polymorphism of genes other than *HLA* and *IFNL3*/4 and the likelihood of spontaneous clearance of HCV infection.

Gene Polymorphism	Region	Participants	OR of Spontaneous Clearance (95% CI)	Reference
***PON1***				
**rs854560** **TT genotype**	Poland	187 hemodialyzed patients	TT *vs*. AA + AT 6.21 (1.96–19.64) MA	[97]
**rs662** **GG genotype**	Poland	187 hemodialyzed patients	GG *vs*. AA + AG 10.762 (1.22- 94.80) MA	
***CD24***				
**rs8734** **CT genotype**	China	622 Chinese (Han population)	CT *vs*. CC 2.11 (1.19–3.73) MA	[96]
***RORC***				
**rs9826** **C allele**	China	137 Chinese women (Han population)	1.97 (1.14–3.41) UA	[102]
**rs1521177** **G allele**	China	137 Chinese women (Han population)	2.18 (1.24–3.82) UA	
***MxA***				
**rs2071430** **TT genotype**	China	1090 Chinese (Han population)	TT *vs*. GG 1.22 (1.01–1.48) MA	[128]

MA—multivariate analysis; OR —odds ratio; UA—univariate analysis.

### 3.4. HCV Genotypes

The HCV genome is highly variable, with six major genotypes (GTs) distinguished so far (GT1-GT7) with numerous subtypes [129]. In 2015, a report was published on the identification of a new GT detected in several patients from the Democratic Republic of Congo, different from the previously known GT1-6, which was classified as the seventh [130]. The classification was expanded to include the new GT8 in 2018 when a new HCV lineage was identified in several patients from Punjab, India [131]. It is uncertain whether this exhausts the topic of HCV genetic diversity, as a newly published report indicates the possibility of discovering another genotype, which has so far been identified in one patient from Guyana [132]. Particular genotypes differ in geographic distribution, clinical significance, and response to antiviral treatment in the era of interferon-based therapies, although currently, all can be effectively treated with pangenotypic DAA regimens [8,133]. Therefore, it is important to consider whether particular GTs may impact SC rates.

GT1 is generally associated with a higher likelihood of SC [9,64,134]. However, GT 3 also shows a high clearance rate, particularly in certain populations, such as young Caucasian men [24]. Information on GT4, GT5, and GT6 is sparse due to their lower prevalence, and for GT7 and GT8, there is no such data because only single infected patients have been identified so far. Direct comparison of SC rates between HCV GTs is challenging as it would require global analysis, encompassing a large number of recruited and followed-up individuals with acute HCV infection. Only prospective observation could answer the question about the impact of the HCV genotype, while the influence of other factors, such as gender, lifestyle, gene polymorphism, or coinfections, can be estimated on the basis of retrospective studies by comparing HCV RNA-positive and negative groups. For this reason, the data are sparse, and the results of the few available analyses tend to be divergent.

In a prospectively observed population of 95 young IVDUs with acute HCV infection and a 21% spontaneous resolution rate, infection with GT3 was associated with a marginally negative association with SC compared with GT1 [19]. Completely different conclusions were drawn by the authors of the analysis assessing the resolution of acute HCV infection in 214 Italian patients; in this study, GT3 was associated with the highest chances of SC compared with GT1, GT2, and GT4 [22]. Similar results were obtained in a German cohort of IVDUs with acute HCV infection; patients infected with GT3 significantly more often experienced SC compared with those infected with GT1 [135], a study from the US involving 67 patients with acute HCV infection, most of whom were IVDUs, did not show that genotype had any association with SC [136]. The largest analyzed population with acute HCV infection was studied in the International Collaboration of Incident HIV and Hepatitis C in Injecting Cohorts (InC^3^) Study, which included nine prospective cohorts of patients from various countries [134]. This study, in turn, documented that in a group of 173 of 632 participants with spontaneous HCV clearance, infection with GT1 was an independent prognostic factor for SC as compared to non-GT1 infection. The role of GT1 infection as a positive predictor of SC was also documented in a single retrospective analysis of this phenomenon in patients chronically infected with HCV, while other studies did not support such observation [33,38].

Therefore, the number of studies reporting the impact of GT on SC, both in the population with acute and chronic HCV infection, is minimal, and their results are inconsistent, preventing the formulation of definitive conclusions. In addition, the genetic polymorphism of the *IFNL4* gene, especially the CC genotype at rs12979860, also plays a crucial role in enhancing SC across different GT of HCV [60,134,137,138]. One should note that GT1 is likely less responsive to interferon treatment compared with other GTs [139]. Therefore, particular variants of *IFNL4* may play a more profound role in its natural course.

### 3.5. Viral Coinfections

The coinfection with some viruses can affect the natural course of HCV infection differently. Hepatitis B virus (HBV) infection, which currently can only be suppressed without complete pathogen elimination [140], may lead to an increased likelihood of SC (Table 7) of HCV due to complex interactions. Although some in vitro observations indicate that HBV and HCV can replicate in the same hepatocytes without interference [141,142], the clinical observations in coinfected patients do not support this claim. The presence of HBV may inhibit HCV replication, leading to a higher rate of HCV clearance. This reciprocal interference on viral replication suggests that HBV can suppress HCV activity, facilitating its clearance [143,144,145]. One should note that there are conflicting results in this regard, with some experimental results showing that there is a limited HBV replication space in the HCV-infected liver [146] and clinical data indicating that, more commonly, HBV replication is suppressed by HCV [147,148,149]. Nevertheless, there is consistent evidence that HBV coinfection results in higher odds of SC of HCV, indicating that plausibly, in some cases, HBV becomes a dominant virus or that other factors are in play, e.g., related to immune responses that would require further elucidation. In addition, it was shown that men with HBV coinfection are more likely to clear HCV compared with women [144]. This gender-specific difference in viral clearance rates highlights the need for further research into the underlying mechanisms.

Moreover, various studies suggest that HIV coinfection reduces the chance of SC of HCV (Table 7). It is known that robust HCV-specific T cell responses, particularly CD4^+^ T cell responses, are associated with SC of HCV. However, these responses are often weak in HIV-coinfected patients, with generally low CD4^+^ levels, correlating with lower clearance rates [152,153,154,155]. On the other hand, the exact threshold under which the SC is significantly less likely cannot be unequivocally set. Some studies demonstrated that the lower the CD4^+^ count, the lower the odds of SC [17,69], while others found no difference [53], did not report it, or had no statistical power to evaluate this hypothesis [54,66,67,91].

However, despite the general effect of HIV infection on the decreased likelihood of spontaneous HCV clearance, this may be further affected by the genetic makeup. CC genotype at rs12979860 of the *IFNL4* gene, which generally increases the odds of SC (Table 5), also increases the possibility of this event in HIV-positive patients [124,156]. Importantly, successful, highly active antiretroviral therapy that normalizes CD4^+^ T cell count and decreases immune activation can also improve the chances of SC of HCV in HIV-infected patients, although this again may mostly relate to individuals with CC genotype at rs12979860 of the *IFNL4* gene [153]. All in all, these findings highlight the need to screen for HCV infection in patients at increased risk of HIV acquisition.

Whether other viral coinfections could impact the SC of HCV remains to be understood. Some data indicates that infection with the Epstein–Barr virus can enhance the replication of HCV [157]. However, whether this can lead to altered chances of SC is currently unknown and would require further research, taking into consideration other latent viruses with the potential for reactivation.

## 4. Conclusions

**Figure 1:**
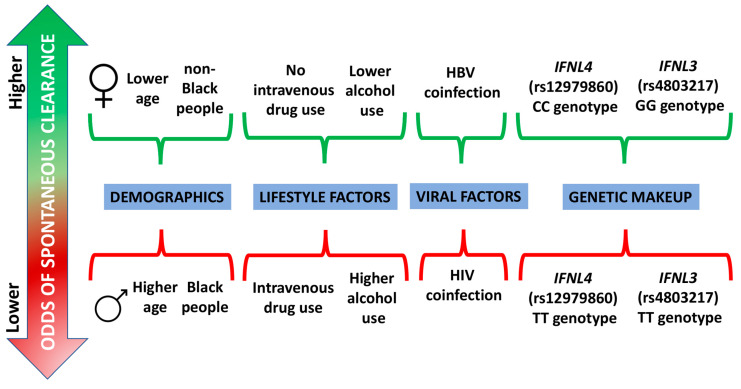
The summary of main factors identified to influence the odds of spontaneous clearance of HCV.

Spontaneous elimination of HCV is a relatively rare phenomenon when compared with rates at which it occurs in the hepatitis B virus infection. It is likely due to a complex combination of demographic, lifestyle, environmental, and genetic factors (Figure 1). However, gene polymorphism, especially rs12979860 of *IFNL4* and rs8099917 of *IFNL3*, as well as certain HLA gene genotypes, may have a leading role in some cases. These may include Keith Richards, guitarist of The Rolling Stones, who reported experiencing SC despite an accumulation of unfavorable factors, i.e., male sex, older age, intravenous and non-intravenous drug use, and alcohol consumption. Understanding the process of SC has multidimensional value. Firstly, it enables clinicians to identify high-risk individuals for improved surveillance, early intervention, and tailored treatments. Secondly, the genetic and immunological underpinnings of SC can inform the development of effective vaccines, authorization of which may put the HCV burden under better control. Thirdly, the molecular mechanisms and host–viral interactions in individuals experiencing SC may open new avenues for targeted therapies that mimic the SC process. Last but not least, recognizing ethic-related differences in SC rates may help address health inequities and guide public health interventions related to the prophylaxis of HCV infection and better access to diagnostics and treatments, particularly in Black people who face persistent disparities in health care and health outcomes. In summary, continued research in the area of SC, particularly elucidating the exact mechanisms behind it, holds promise for reducing the global burden of HCV and improving patient outcomes.

## Figures and Tables

**Table 7:** The summary of analyses evidencing significant associations between viral coinfections and the likelihood of spontaneous clearance of HCV infection.

Region	Participants	OR of Spontaneous Clearance (95% CI)	Reference
**HIV**			
**USA**	*n* = 919 aged ≥ 17	0.33 (0.14–0.80) MA	[17]
**USA**	*n* = 496 mean age 53.5	0.37 (0.16–0.83) MA	[91]
**USA**	*n* = 101 aged ≥ 18	0.41 (0.26–0.65) MA	[53]
**USA**	*n* = 1032 aged ≥ 18	0.76 (0.50–1.15) MA	[69]
**China**	*n* = 347 mean age 27	0.25 (0.07–0.92) MA	[54]
**Canada**	*n* = 762 mean age 42	0.58 (0.38–0.88) UA	[66]
**USA**	*n* = 320 aged ≥ 18	0.48 (0.13–1.73)	[67]
**HBV**			
**USA**	*n* = 919 aged ≥ 17	2.75 (1.00–7.59) MA	[17]
**China**	*n* = 410 mean age 51	5.51 (2.42–12.52) MA	[62]
**China**	*n* = 1918 mean age 36	7.15 (9.51–28.80) MA	[150]
**Taiwan**	*n* = 287 mean age 62	2.37 (1.06–5.26)	[151]
**China**	*n* = 498 median age 34	4.17 (1.88–9.25) MA	[59]
**China**	*n* = 347 mean age 27	8.43 (3.64–19.52) MA	[54]
**USA**	*n* = 496 median age 53.5	5.0 (1.26–28.6) UA	[91]
**USA**	*n* = 101 aged ≥ 18	1.53 (0.90–2.58) UA	[53]
**Europe, Israel, Argentina**	*n* = 1940 median age 37.2	2.91 (1.94–4.38) MA	[64]

MA—multivariate analysis; OR —odds ratio; UA—univariate analysis; MA.

## Data Availability

No new data were created or analyzed in this study.
